# Evaluation of the Clinicopathological Features Associated With Malignancy of Phyllodes Tumor of the Breast

**DOI:** 10.7759/cureus.76221

**Published:** 2024-12-22

**Authors:** Dhierin R Jagdewsing, Ghulam Murtaza, Sima A Jagdewsing, Shruti A Jagdewsing, Noor Safra C Fahmy, FHNS Anthony Silva, Tanul Koendjbiharie, Sherilyn Djojomoenawi, Omane V Kwakye, NM Motachim Mahmud

**Affiliations:** 1 Department of Colorectal Cancer Surgery, Dalian Medical University, Dalian, CHN; 2 Department of Surgery, Services Hospital Lahore, Lahore, PAK; 3 Department of Neurology, Curaçao Medical Center (CMC), Willemstad, CUW; 4 Department of Clinical Medicine, Anton de Kom University, Faculty of Medical Sciences, Paramaribo, SUR; 5 Department of Clinical Medicine, Dalian Medical University, Dalian, CHN; 6 Department of Breast Surgery, Tongji Hospital, Shanghai, CHN; 7 Department of Medicine, ASEAB (Association for Socio-Economic Advancement of Bangladesh) Community Hospital and Diagnostic Center, Pabna, BGD

**Keywords:** breast tumor, clinicopathological features, fibro-epithelial lesion, phyllodes tumor, tumor size and shape

## Abstract

Objective: Phyllodes tumor (PT) is a variant of fibroepithelial proliferations of the breast, histologically demonstrating a leaf-like pattern. The WHO has categorized PTs as benign, borderline, or malignant based on their histological characteristics. The objective of this paper is to assess the clinicopathological factors with malignancy in PT of the breast.

Method: Medical records of 101 diagnosed PT patients in the Second Affiliated Hospital of Dalian Medical University between 2008 and 2023 were reviewed. Information on clinical presentation and histopathological findings of the lesions were retrieved from patient files and/or histological reports, respectively.

Results: Of the 101 patients, all were female and had a mean age of 44.35 ± 14.14 years and mean tumor size of 8.3 ± 5.8 cm The distribution for the histological type was benign (n = 54, 53.4%), borderline (n = 36, 35.6%) and malignant (n = 11, 10.8%). Most benign PTs were observed in younger patients, while borderline and malignant PTs involved elderly patients, with a mean age of 47.56 ± 11.86 years for borderline PT and a mean age of 46.55 ± 11.62 years for malignant PT. Benign PTs had a mean size of 5.58 ± 2.29 cm, while those of borderline and malignant were larger, with a mean size of 10.58 ± 6.79 cm and 14.90 ± 6.44 cm, respectively. Malignant PTs had higher lactate dehydrogenase (LDH) levels of 232 ± 91.5 U/L compared to borderline PTs, 177.9 ± 19.9 U/L, and benign PTs, 177.6 ± 39.9U/L. The course of the disease of the malignant PT group was slightly longer (436.9 ± 391.3 weeks) than that of the benign (44.17 ± 71.54 weeks) and borderline (54.33 ± 94.33 weeks). In histopathology, necrosis was observed only in malignant PTs (81.8%), and severe stromal atypia was seen in 72.7% of malignant cases. The mitotic count was highest in malignant PTs at 13.18 ± 4.43 HPF as compared to benign 3.52 ± 2.97 HPF and borderline PTs at 7.28 ± 2.21 HPF.

Conclusion: Benign PTs were more common in this study than malignant or borderline PTs. There was a highly significant correlation between patient age, tumor size, LDH, and disease progression in all subtypes of PT. This analysis showed that malignant PTs were larger and observed in older patients with higher LDH and with a longer duration of the disease. Other factors, in addition to histological properties, are useful in determining PT behavior and management. More studies at an advanced level of evidence in the form of randomized trials are required when developing a risk classification for PT based on patient age, tumor size, and LDH.

## Introduction

Breast cancer is the most common cancer among women worldwide, with an estimated 2.3 million new cases diagnosed annually [1]. Phyllodes tumors (PTs) are rare fibroepithelial neoplasms of the breast and contribute to less than 1% of breast malignancies [2,3]. Phyllodes tumors can exhibit both benign epithelial and malignant stromal components. While PTs can grow to substantial sizes, sometimes reaching 30-40 cm in size [4], axillary lymph node metastases are unusual (occurring in less than 3%), and distant metastases most frequently involve the lung (approximately 66%) and bones (around 28%) [5,6]. PT often manifests as a mass without pain, involving mobility with defined margins [7].

PTs have not been found to have specific definitive risk factors. Abnormalities, including +1q and +5p, are seen in borderline and malignant types, and women with Li-Fraumeni syndrome are prone to this condition. Compared to other populations, Asian women are often diagnosed at a younger median age of 45 years [8]. The WHO divides PTs into benign and borderline PTs with malignant features based on cellularity, tumor margins, mitotic frequency, and stromal pleomorphism. Thus, benign PTs comprise 58.4-74.6 % of all cases with 10-17% recurrence rates and 9.3-31% of malignant PTs with local recurrence of cases (23-30%) and metastases [9,10].

## Materials and methods

This was a retrospective cohort study conducted at the Breast Surgery Department of the Second Affiliated Hospital of Dalian Medical University, Dalian, China. A total of 101 PTs diagnosed and recorded between 2008 and 2023 were included in the study. Collected data included the patient’s age at the time of diagnosis, the stage of the disease, tumor size, family/personal health history, initial complaint and symptoms, tumor location, mammographic findings, prior clinical diagnosis, surgical treatments, histology of the tumor, and prognosis. Qualitative data from clinical records and patients, departmental clinical databases, and histopathological records were collected.

The inclusion criteria for this study were patients diagnosed with PT based on histopathological examination and complete clinical and histopathological data available from the medical records. Patients of all ages and tumor stages (benign, borderline, and malignant) were included to ensure comprehensive data collection. The exclusion criteria were incomplete clinical or histopathological records, concurrent diagnoses of other breast malignancies, prior treatment for breast cancer, and patients with missing or unclear follow-up data. These criteria ensured a focused and reliable analysis of clinicopathological characteristics associated with PT stages while maintaining the integrity of the dataset.

Data analysis was done using the IBM SPSS Statistics for Windows, Version 29.0.2 (Released 2023; IBM Corp., Armonk, New York, United States). Comparisons in PT stages of continuous variables, including benign, borderline, and malignant, were tested using one-way analysis of variance (ANOVA). Descriptive categorical data analysis was done by displaying the frequencies and the percentages, given that most of the data fell under this type of variable. The correlations between clinicopathological characteristics and PT stages were analyzed with Pearson’s chi-squared test. The statistical significance was determined such that if the calculated probability showed the likelihood of data occurrence was less than 0.05, then it was accepted. This approach allowed for the assessment of both continuous and categorical data, thus allowing the complete assessment of clinicopathological associations with PT stages. Data were analyzed using descriptive statistics (mean ± SD) for continuous variables and frequencies with percentages for categorical variables.

## Results

In Table 1, the clinicopathological characteristics of 101 female PT cases can be seen enumerated by tumor types: 53.4% benign, 35.6% borderline, and 10.8% malignant. The patients were evenly split over 5-year age bands, with 52.5% of the patients being aged between 31 and 50 years old and the average age of patients being 44.35 ± 14.14 years. Overall, tumor size was 8.3 ± 5.8 cm; 51.5% of tumors were between 5-10 cm. The mitoses/ HPF was 5.91 ± 4.4, and the disease duration was 90.56 ± 18.96 weeks. Resected operations included radical mastectomy and sentinel lymph node exploration (23.8%), breast-conserving surgery (69.3%), and ultrasound-guided minimal invasive surgery (6.9%). Necrosis was shown in 8.9% of cases. Stromal hyperplasia was detected in 49.5% of tumors, and stromal atypia was rated mild in 67.3%, moderate in 24.8%, and severe in 7.9%.

**Table 1:** Clinicopathological features of phyllodes tumor patients (N=101)

Clinicopathological Features	Values
Phyllodes Tumor Types	
Benign, n (%)	54 (53.4)
Borderline, n (%)	36 (35.6)
Malignant, n (%)	11 (10.8)
Age (Years), mean ± SD	44.35 ± 14.14
Female, n (%)	101 (100)
Age Groups	
≤ 30 years, n (%)	16 (15.8)
31-50 years, n (%)	53 (52.5)
> 50 years, n (%)	32 (31.7)
Size of the Tumor (cm), Mean ± SD	8.3 ± 5.8
Tumor Size Groups	
<5 cm, n (%)	24 (23.8)
5-10 cm, n (%)	52 (51.5)
>10 cm, n (%)	25 (24.8)
Number of Mitoses (/HPF), mean ± SD	5.91 ± 4.4
Course of Disease, mean ± SD	90.56 ± 18.96
Surgery Performed	
Radical mastectomy and sentinel lymph node exploration, n (%)	24 (23.8)
Breast-conserving surgery, n (%)	70 (69.3)
Ultrasound-guided minimal invasive surgery, n (%)	7 (6.9)
Necrosis, n (%)	9 (8.9)
Stromal hyperplasia, n (%)	50 (49.5)
Stromal Atypia	
Mild, n (%)	68 (67.3)
Moderate, n (%)	25 (24.8)
Severe, n (%)	8 (7.9)

Table 2 highlights the clinicopathological profile in patients with benign, borderline, and malignant tumors. In this table, statistical analyses were performed using one-way ANOVA for continuous variables and Pearson’s chi-squared test for categorical variables. Patients with borderline and malignant tumors are significantly older than those with benign tumors, and malignant tumors are confirmed to have a significantly prolonged duration of disease, larger size, and greater circumference compared with borderline cases (p < 0.05). The mitotic rate is significantly higher in malignant tumors (p < 0.001), and lactate dehydrogenase (LDH) is also significantly elevated. As for the operations, marginal mastectomy is performed in cases where the tumor is not definitely malignant, while surgical intervention for cancerous tumors involves breast-conserving therapy. Necrosis occurs only in malignancies, but fibrosis is seen in benign conditions, characterizing a significant pathological difference between groups (P < 0.003). Moreover, in all borderline cases, pain and tenderness were identified as the most common complaints (p = 0.015); however, there were no differences in terms of menstrual cycle or marital status between the groups. Malignant tumors are developed by the slow growth of the tumor bulk; however, statistically, this is insignificant. Subgroup analysis of the surface ultrasound reveals that there is no large difference in the distribution of solid nodules. The recurrence rate of malignant tumors is slightly higher, but the difference is not statistically significant.

**Table 2:** Clinicopathological characteristics and phyllodes tumor subtypes LDH: lactate dehydrogenase

Clinicopathological Features	Benign (n=54)	Borderline (n=36)	Malignant (n=11)	P-value
Age (years), mean ± SD	41.76 ± 15.62	47.56 ± 11.86	46.55 ± 11.62	0.003
Course of disease (weeks), mean ± SD	44.17 ± 71.54	54.33 ± 94.33	436.9 ± 391.3	<0.001
Size of tumor (cm), mean ±SD	5.58 ± 2.29	10.58 ± 6.79	14.90 ± 6.44	<0.020
Tumor circumference (cm^2^), mean± SD	27.4 ± 24.2	114.6 ± 147.1	227.6 ± 185.9	<0.001
Number of mitoses / HPF, mean ± SD	3.52 ± 2.97	7.28 ± 2.21	13.18 ± 4.43	<0.001
LDH (U/L), mean ± SD	177.6 ± 39.9	177.9 ± 19.9	232 ± 91.5	<0.001
Surgery Performed				<0.001
Radical mastectomy sentinel lymph node exploration, n (%)	3 (5.6)	19 (52.8)	2 (18.2)	
Breast-conserving surgery, n (%)	46 (85.2)	15 (41.7)	9 (81.8)	
Ultrasound-guided minimally invasive surgery, n (%)	5 (9.3)	2 (5.6)	0 (0.00)	
Necrosis, n (%)	0 (0)	0 (0)	9 (81.8)	<0.001
Stromal Atypia				
Mild, n (%)	54 (100)	14 (38.9)	0 (0.00)	<0.001
Moderate, n (%)	0 (0)	22 (61.1)	3 (27.3)	<0.001
Severe, n (%)	0 (0)	0 (0)	8 (72.7)	<0.001
Stromal Hyperplasia, n (%)	25 (46.3)	18 (50.0)	11 (100)	0.576
Pain and Tenderness, n (%)	7 (13.0)	14 (38.9)	2 (18.2)	0.015
Menstrual Cycle status				0.208
Regular menstrual cycle, n(%)	46 (85.2)	23 (63.9)	9 (81.8)	
Irregular menstrual cycle, n (%)	3 (5.6)	6 (16.7)	1 (9.1)	
Menopause, n (%)	5 (9.3)	7 (19.4)	1 (9.1)	
Marital Status				0.378
Married, n (%)	46 (85.2)	32 (88.9)	11 (100)	
Unmarried/Divorced/Widowed, n (%)	8 (14.8)	4 (11.1)	0 (0)	
Gradual increase in tumor size				0.062
Present, n (%)	20 (37.0)	19 (52.8)	8 (72.7)	
Absent, n (%)	34 (63.0)	17 (47.2)	3 (27.3)	
Ultrasound Findings, n (%)				0.393
Right breast solid nodules	32 (59.3)	15 (41.7)	5 (45.5)	
Left breast solid Nodules	10 (18.5)	9 (25.0)	4 (36.4)	
Bilateral breast solid nodules	12 (22.2)	12 (33.3)	2 (18.2)	
Number of Tumor nodules				0.691
Single tumor nodules, n (%)	33 (61.1)	21 (58.3)	8 (61.4)	
Multiple tumor nodules, n (%)	21 (38.9)	15 (41.7)	3 (27.3)	
Recurrence, n (%)	6 (11.1)	2 (5.6)	3 (27.3)	0.129
Necrosis, n (%)	0 (0)	0 (0)	9 (81.8)	<0.001
Stromal Atypia				<0.001
Mild, n (%)	54 (100)	14 (38.9)	0 (0.00)	
Moderate, n (%)	0 (0)	22 (61.1)	3 (27.3)	
Severe, n (%)	0 (0)	0 (0)	8 (72.7)	
Stromal Hyperplasia				0.576
Present, n (%)	25 (46.3)	18 (50.0)	11 (100)	
Absent, n (%)	29 (53.7)	18 (50.0)	0 (0.00)	

**Figure 1:**
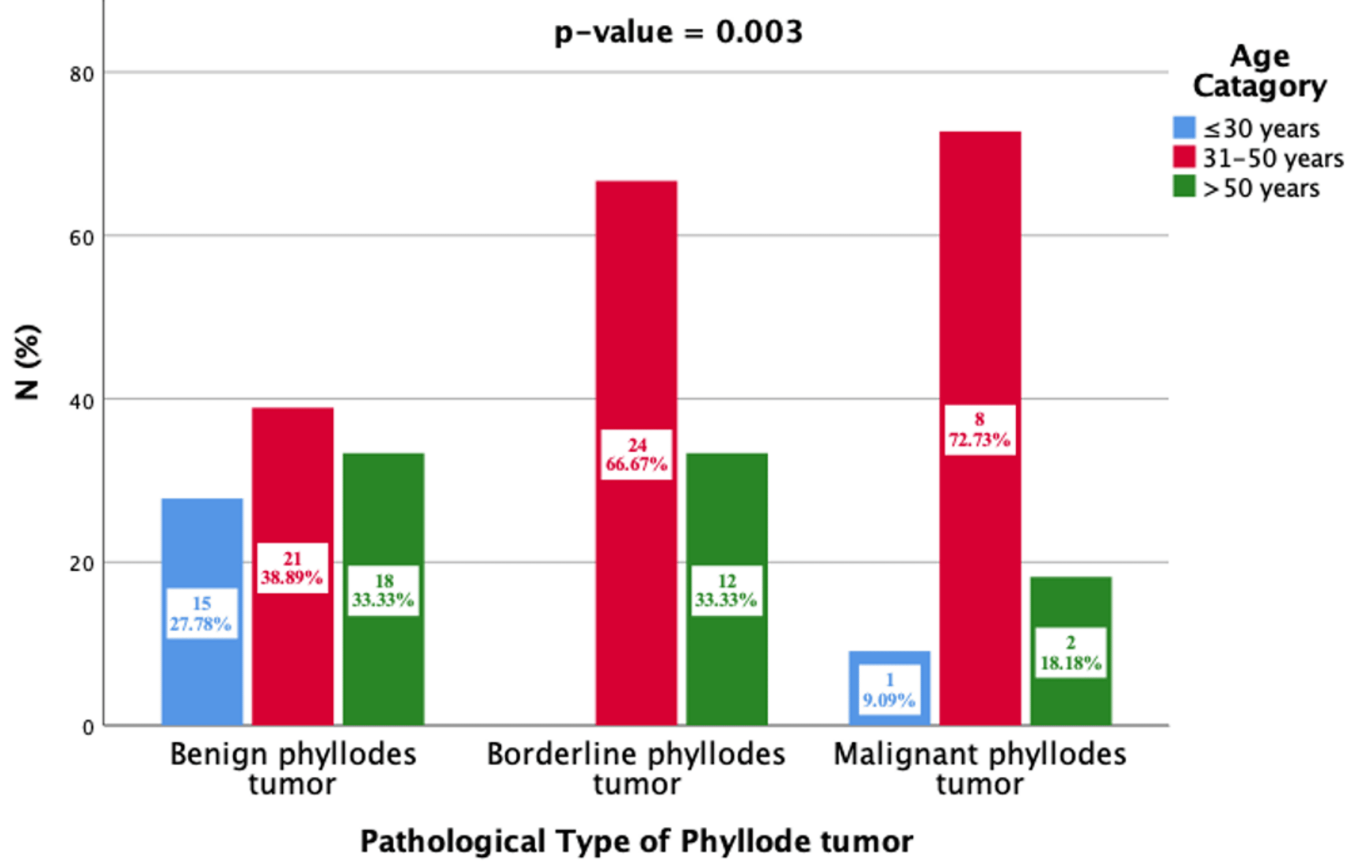
Association of phyllodes tumor types with age group (N=101)

Figure 1 illustrates the age distribution of patients with benign, borderline, and malignant PTs, categorized into three age groups: ≤30 years, 31-50 years, and >50 years. Statistical analyses were performed using Pearson’s chi-squared test, which showed a p-value of 0.003 (statistically significant). As to the age distribution of the tumor types, the age range between 31 and 50 shows the highest percentage of cases, which means that the middle-aged population is the most affected by the disease. Benign tumors occur at all ages with moderate increment in the 31-50 years and >50 years age groups. Most of the borderline tumor cases are diagnosed in the 31-50 years age group, while the >50 years age group has far fewer cases, and the ≤30 years group has very few cases. Similarly to benign neoplasms, malignant tumors are also most diagnosed in patients aged 31-50 years and >50 years, with relatively few patients under the age of 30. Thus, these results highlight the fact that even though PTs can develop at any age, borderline and malignant ones are more typical in middle-aged and elderly women. This goes to show the necessity of practicing targeted surveillance and accurate early diagnosis for patients in the 31-50 years age range.

**Figure 2:**
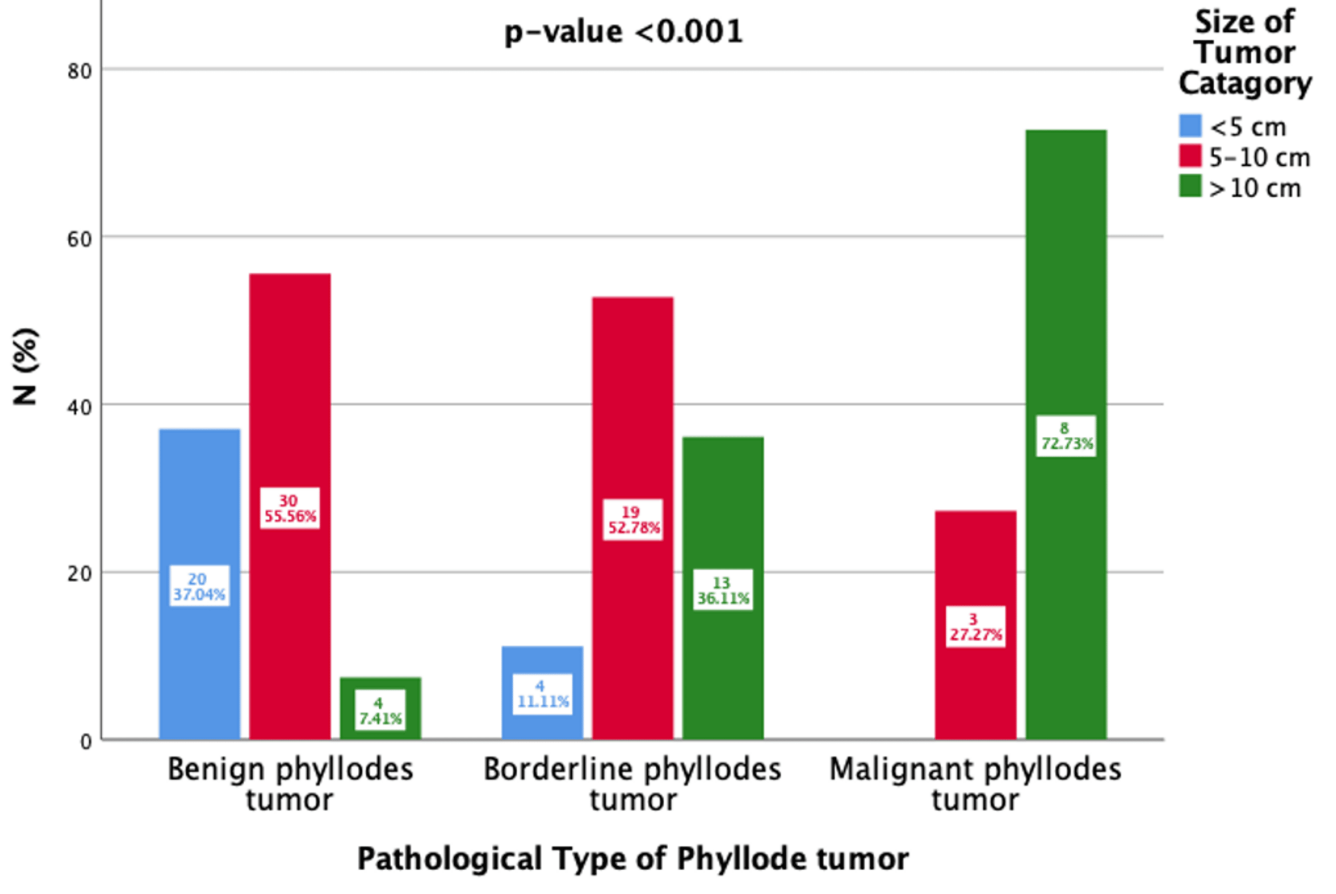
Association of phyllodes tumor types with tumor size (N=101)

Figure 2 illustrates the distribution of PTs (benign, borderline, and malignant) based on tumor size categories. Statistical analyses were performed using Pearson’s chi-squared test, which showed a p-value of <0.001 (statistically significant). By size, the majority of benign PTs are <5 cm, with moderate occurrences in the 5-10 cm range and very few in the >10 cm size range, suggesting that smaller tumors are more likely to be benign. Most of the tumors were found to be of borderline size in the group 5-10 cm, and an even smaller percentage of tumors were found in the less than 5 cm and more than 10 cm groups, which indicates that tumors with intermediate size are likely to be borderline tumors. Malignant tumors are mainly found in the >10 cm, followed by 5-10 cm, and <5 cm was the least reported. Based on such observation, it is possible to say that tumor size is an indicator of malignancy risk; more specifically, small tumors are unlikely to be malignant, while large tumors are considered malignant. From a clinical perspective, this has emphasized the need for early tumor detection and follow-up since larger (> 10 cm) tumors may call for invasive diagnostic/ therapeutic procedures. Tumor size should, therefore, become a parameter for diagnostics and treatment algorithms.

## Discussion

PTs are rare fibroepithelial neoplasms of the breast, accounting for less than 1% of breast tumors, and they are most commonly diagnosed in women between the ages of 40 and 50 years, with a peak incidence around 45-49 years [11]; however, in Asian populations, there is a comparatively younger age group for the disease [12,2]. Initially, PTs were first characterized by Johannes Müller in 1838 [9]. They were later found to possess malignant potential, and metastasis examples were reported as early as 1931 [13]. Benign PT accounts for 58.4-74.6%, while malignant PS has higher rates of recurrence and metastasis [6]. Histologically, benign PTs have low mitotic activity and mild nuclear atypia; however, malignant PTs have severe stromal atypia and necroses. This emphasizes the significance of thorough histological examination for predicting the tumor‘s potential behavior and guiding appropriate management strategies [6,14]. In our study, benign PTs constituted 53.4%, borderline PTs 35.6%, and malignant PTs 10.9%, which is in line with other studies that showed benign PT ranging from 35-85%, borderline from 7-40%, and malignant from 7-45% [2,15]. Younger women are more likely to develop benign PTs, while borderline and malignant PTs are more prevalent in older patients. A correlation between tumor size and malignancy can be seen where benign PTs have a smaller size (5.58 ± 2.29) compared to borderline and malignant PTs. Also, LDH levels were higher among patients with malignant PT (232 ± 91.5 U/L) as compared to borderline and benign PT (177.9 ± 19.9 U/L) and (177.6 ± 39.9 U/L) (p < 0.001).

Clinically, PTs typically present as firm, round, painless nodules, often located in the upper outer quadrant of the breast. The growth rate and size of the tumor can indicate malignancy. If the areola is involved, the nipple may retract or discharge blood at times. This research showed an overall average tumor size of 8.3 ± 5.8 cm as compared with 4-5 cm observed in other studies, potentially attributable to the Asian ethnicity of most of the patients in our study [2,16]. Clinically, PTs are often firm and round, painless nodules whose size and growth rate can predict malignancy. Combined clinical imaging and histopathological diagnostic modalities are ideal for diagnosing and managing these diseases [17]. Radiological imaging is essential in evaluating PTs, and mammography presents well-circumscribed masses that may have calcifications [18]. Age at diagnosis influences prognosis since breast cancer patients diagnosed later in life have more aggressive PTs. Cancer size, persistence time, some histological characteristics of the tumor tissue, such as stromal atypia and necrosis, and the patient’s symptoms are connected to prognosis; pain often points to the aggressiveness of the process [19,20]. PTs often coexist with fibroadenomas in 4.2-33.3% of cases [21]. Imaging modalities such as mammography and ultrasound can reveal well-defined masses, but these are not always conclusive in distinguishing PTs from fibroadenomas. PTs appear as hypoechoic solid masses with posterior acoustic enhancement on ultrasound and heterogeneously enhancing masses on MRI. Malignant PTs may show abnormal enhancement and dense MRI signal contrast [22]. CT scans typically reveal well-defined, dense masses; however, imaging characteristics alone cannot reliably differentiate between benign, borderline, or malignant PTs [23].

Histopathological examination remains crucial for diagnosis and prognostication. PTs are biphasic tumors primarily composed of hypercellular stroma, cleft-like spaces, and leaf-shaped patterns [14]. Benign PTs have low mitotic figures and mild stromal atypia, whereas malignant PTs have high mitotic activity and necrosis [6]. Immunohistochemical (IHC) markers such as Ki-67, p53, and CD117 (c-kit) provide valuable insight into the biological activity of PTs. It is a valuable tool in assessing the biological activity of PTs of the breast. The Ki-67 labeling index, which measures cellular proliferation, tends to be higher in more malignant PTs. However, studies have shown that Ki-67 does not consistently correlate with tumor recurrence. Therefore, while Ki-67 can provide insights into tumor aggressiveness, it should not be solely relied upon to predict recurrence in PTs [24,25]. Overexpression of p53 indicates disrupted cell cycle regulation and malignant activity [26]. Only about 40% of benign PTs are positive for CD117 (c-kit) or CD34 compared to n90% of malignant PTs [27]. Other biomarkers useful in the subclassification of PT include CD10, which is higher in malignant cases, and reduced Bcl-2 levels suggest aggressive tumors [28,29]. Additionally, estrogen and progesterone receptor expression donates a more favorable biology of the tumor, while vascular endothelial growth factor (VEGF) promotes tumor progression through angiogenesis [30].

Concerning the surgical treatment, breast-conserving surgery was more frequent in benign (85.2%) and malignant (81.8%) PT, while radical mastectomy and sentinel lymph node excision were more common in borderline PT (52.8%). There was a statistically significant difference in the surgical approach (p < 0.001). In the present study, malignant PT had a relatively longer disease duration in weeks 436.9± 391.3 weeks) than benign (44.17 ± 71.54 weeks) PT as well as borderline PT (54.33± 94.33 weeks) (p < 0.001). Histologically, we observed a higher mitotic count in malignant PT, which was 13.18 ± 4.43/HPF, than borderline 7.28 ± 2.21/HPF and benign 3.52 ± 2.97/HPF (p < 0.001). Stromal atypia and stromal hyperplasia were noted more often in malignant PT. Based on our findings, clinicopathological variables such as the size of the tumor, mitotic count, stromal atypia, and level of LDH are significant for the behavior and prognosis of PT. PT recurrence rates were low in our study, and previous studies also reported rare PT recurrence if PT was diagnosed early and if timely adequate surgical intervention was performed.

However, the following limitations should be noted. First, because the study design is retrospective, there might be some limitations due to missing data or data incompleteness. Second, the sample size for malignant PTs is comparatively small, and the power of statistical testing performed in this study is therefore low, and the conclusion for this specific group of patients cannot be overemphasized. Third, this was a single-center study, and the population was predominantly Asian, so we may not have captured the larger ethnic differences that are seen around the world. Lastly, due to the absence of long-term follow-up data, there was a limitation in the assessment of recurrence rates as well as the survival of patients. Large-scale, multicenter, perhaps randomized control trials with longer follow-ups should be conducted to confirm such results.

## Conclusions

The present study presented a clinicopathological analysis of PT of the breast along with a discussion of its basic characteristics depending on the benign, borderline, and malignant tumor. The results emphasize stronger positive relations of tumor size, patients’ age, LDH, disease duration, and histopathological markers that determine the tumor behavior and prognosis. Malignant PT was, in particular, more aggressive with higher frequencies of large tumor size, high mitotic activity, necrosis, and severe stromal atypia, which correlated to higher age and PT duration. These differences stress the significance of the inclusion of these parameters into the clinical risk assessment for better diagnosis and treatment. Benign PT was more frequently observed and was characterized by a less aggressive histological profile; however, our findings emphasize the importance of careful examination of patients with borderline disease for identification of those with a potential high risk for disease progression and recurrence. The low recurrence level documented in the current study affords confidence in surgical management but underlines the importance of surveillance in averting late syndromes, especially in malignant diseases.

This work opens the door to more detailed investigation in the future into the genetic and molecular causes and effects of PT to identify new targets for treatment and potential biomarkers. Through further analysis of chromosomal abnormalities, gene profiling, and molecular signaling, future research may discover molecular markers that enhance the sensitivity of screening as well as define tailored treatment regimens. In terms of an interdisciplinary approach, it is crucial that surgeons, pathologists, and oncologists come together to achieve the best outcomes for their patients. Regarding new applications of technology, areas like artificial intelligence and machine learning have the potential to improve not only risk assessment but also provide implications for the management of patients. Therefore, large-scale, multicenter clinical, and randomized trials incorporating our findings are required in different populations. By bridging current gaps in knowledge and refining our approach to PT management, this study contributes to a broader understanding of these rare tumors and advances the field toward more effective and individualized patient care.
